# 10-(6-Hy­droxy­hexa-2,4-diyn-1-yl)-10*H*-phenothia­zine 5-oxide

**DOI:** 10.1107/S1600536812027511

**Published:** 2012-06-27

**Authors:** Hideyuki Tabata, Tsunehisa Okuno

**Affiliations:** a Department of Material Science and Chemistry, Wakayama University, Sakaedani, Wakayama 640-8510, Japan

## Abstract

The title compound, C_18_H_13_NO_2_S, has two independent mol­ecules (*A* and *B*) with similar conformations in the asymmetric unit. Both phenothia­zine moieties have a butterfly structure [dihedral angles between benzene rings = 155.17 (7) and 161.71 (7)°, respectively], and the central six-membered rings have a boat form. In the crystal, the *A* and *B* mol­ecules stack alternately along the *b* axis. The *A* and *B* mol­ecules are linked by O—H⋯O=S hydrogen bonds, forming zigzag chains along [10-1].

## Related literature

For related structures of phenothia­zine 5-oxide compounds, see: Chu *et al.* (1985 ▶); Dahl *et al.* (1982 ▶); Hough *et al.* (1985*a* ▶,*b* ▶, 1982 ▶); Jin *et al.* (2010 ▶); Jovanovic *et al.* (1986 ▶); Okuno *et al.* (2006 ▶); Wang *et al.* (2009 ▶); Xu *et al.* (2009 ▶). For the related preparation of 10-(6-hy­droxy­hexa-2,4-diyn-1-yl)-10*H*-pheno­thia­zine, see: Zaugg *et al.* (1958 ▶) and for the preparation of the title compound, see: Gilman & Ranck (1958 ▶).
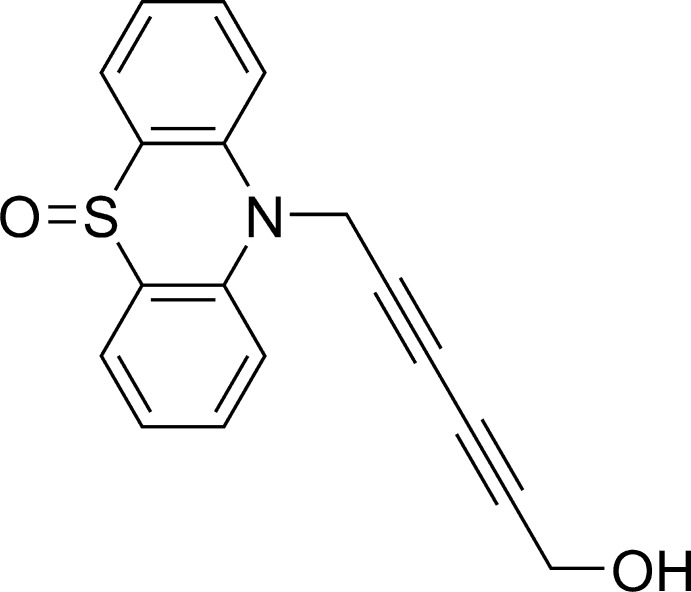


## Experimental

### 

#### Crystal data

C_18_H_13_NO_2_S
*M* *_r_* = 307.37
Monoclinic, 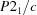

*a* = 16.797 (5) Å
*b* = 10.197 (3) Å
*c* = 17.664 (5) Å
β = 94.934 (5)°
*V* = 3014.3 (15) Å^3^
*Z* = 8
Mo *K*α radiation
μ = 0.22 mm^−1^
*T* = 93 K
0.15 × 0.15 × 0.05 mm

#### Data collection

Rigaku Saturn724+ diffractometer
Absorption correction: numerical (*NUMABS*; Rigaku, 1999 ▶) *T*_min_ = 0.969, *T*_max_ = 0.989
24445 measured reflections
6932 independent reflections
5523 reflections with *F* ^2^ > 2σ(*F* ^2^)
*R*_int_ = 0.033

#### Refinement

*R*[*F* ^2^ > 2σ(*F* ^2^)] = 0.050
*wR*(*F* ^2^) = 0.135
*S* = 1.08
6931 reflections
404 parameters
1 restraint
H atoms treated by a mixture of independent and constrained refinement
Δρ_max_ = 0.88 e Å^−3^
Δρ_min_ = −0.47 e Å^−3^

### 

Data collection: *CrystalClear* (Rigaku, 2008 ▶); cell refinement: *CrystalClear*; data reduction: *CrystalClear*; program(s) used to solve structure: *SHELXS97* (Sheldrick, 2008 ▶); program(s) used to refine structure: *SHELXL97* (Sheldrick, 2008 ▶); molecular graphics: *ORTEP-3* (Farrugia, 1997 ▶); software used to prepare material for publication: *CrystalStructure* (Rigaku, 2010 ▶).

## Supplementary Material

Crystal structure: contains datablock(s) global, I. DOI: 10.1107/S1600536812027511/ff2072sup1.cif

Structure factors: contains datablock(s) I. DOI: 10.1107/S1600536812027511/ff2072Isup2.hkl

Supplementary material file. DOI: 10.1107/S1600536812027511/ff2072Isup3.cml

Additional supplementary materials:  crystallographic information; 3D view; checkCIF report

## Figures and Tables

**Table 1:** Hydrogen-bond geometry (Å, °)

*D*—H⋯*A*	*D*—H	H⋯*A*	*D*⋯*A*	*D*—H⋯*A*
O2—H13⋯O3^i^	0.82 (3)	1.85 (3)	2.663 (3)	172 (3)
O4—H26⋯O1^ii^	0.85 (2)	1.81 (2)	2.659 (3)	175 (3)

Symmetry codes: (i) 
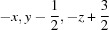
; (ii) 
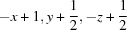
.

**Fig. 1.**
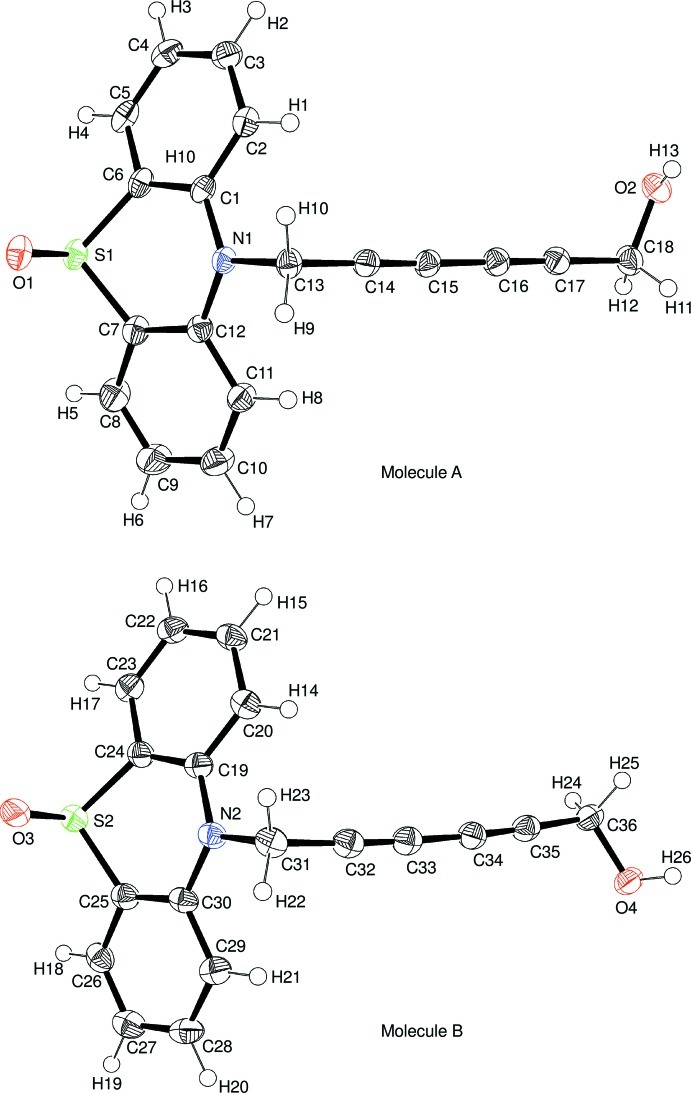
The asymmetric unit of the title compound with atom-numbering scheme. Displacement ellipsoids are drawn at the 50% probability level and H atoms are shown as small spheres.

**Fig. 2.**
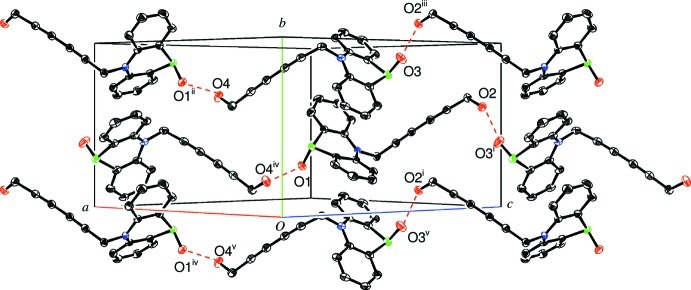
A view of the two-dimensional array of the title compound on the (101) plane. Hydrogen bonds are shown as dashed lines, and hydrogen atoms are omitted for clarity. [Symmetry codes: (i) -x, y - 1/2, -z + 3/2; (ii) -x + 1, y + 1/2, -z + 1/2; (iii) -x, y + 1/2, -z + 3/2; (iv) -x + 1, y - 1/2, -z + 1/2; (v) x, y - 1, z].

## supplementary crystallographic information

### Comment

Aromatic compounds that contain S-atom in a substituent and/or within an aromatic ring have attracted interest from the viewpoint of electronic property of the compounds. Oxidation of S-atom to form S=O bond enables to control its electronic condition without remarkable structural changes. S=O bonds have also been paid attention due to their ability to control the molecular arrangements.

In the title compound, there are two independent molecules (A and B) in the unit cell (Figure 1). The molecular structures of A and B are similar. The phenothiazine moieties have a butterfly structure, where the dihedral angles between two benzene rings (the C1—C6 plane: r.m.s. deviation = 0.0114 Å and the C7—C12 plane: r.m.s. deviation = 0.0020 Å in A, the C19—C24 plane: r.m.s. deviation = 0.0033 Å and the C25—C30 plane: r.m.s. deviation = 0.0052 Å in B) are 155.17 (7)° and 161.71 (7)°, respectively. The central six-membered rings (the N1/C1/C6/S1/C7/C12 and the N2/C19/C24/S2/C25/C30 rings) have a boat form. The S1—O1 and S2—O3 bonds showed longer bond lengths compared with the reported values (1.434 (13) Å - 1.511 (3) Å) of phenothiazine 5-oxide compounds. (Chu *et al.*, 1985; Dahl *et al.*, 1982; Hough *et al.*, 1982; Hough *et al.*, 1985*a*; Hough *et al.*, 1985*b*; Jin *et al.*, 2010; Jovanovic *et al.*, 1986; Okuno *et al.*, 2006; Wang *et al.*, 2009; Xu *et al.*, 2009). The elongation might be caused by the intermolecular hydrogen bonds.

The A and B stack alternately along the *b* axis. There are not any remarkable contacts within the stacks. The A and B molecules are connected by O—H···O=S hydrogen bonds, forming zig-zag chains along the [101] direction, where the distances of O2···O3^i^ and O4···O1^ii^ [Symmetry codes: (i) -*x*, *y* - 1/2, -*z* + 3/2; (ii) -*x* + 1, *y* + 1/2, -*z* + 1/2] are 2.663 (3) Å and 2.659 (3) Å, respectively (Figure 2). In this compound, S=O bonds play an important role to link the stacks by the intermolecular hydrogen bonds.

### Experimental

10-(6-Hydroxyhexa-2,4-diyn-1-yl)-10*H*-phenothiazine

*N*^1^,*N*^1^,*N*^4^,*N*^4^-Tetramethylethylenediamine (TMEDA; 30 µl, 0.20 mmol) was added to a suspension of copper(I) chloride (57 mg, 0.58 mmol) in degassed acetone (4 ml), and the suspension was stirred for 30 min. The supernatant solution containing the CuCl-TMEDA catalyst was transferred to a solution of 10-(prop-2-yn-1-yl)-10*H*-phenothiazine (0.67 g, 2.82 mmol) (Zaugg *et al.*, 1958) and 2-propyn-1-ol (1.6 ml, 28 mmol) in acetone (3 ml). The solution was stirred for 6 days under an oxygen atmosphere. After the concentration of the solution, the residue was extracted with dichloromethane (20 ml). The solution was washed with 0.5 *M* aqueous hydrogen chloride (7 ml) and water (20 ml × 3) successively. The water layer was extracted twice with dichloromethane (200 ml). After the concentration of the combined solution, the residue was purified by a recrystallization from a *n*-hexane to give a 10-(6-hydroxyhexa-2,4-diyn-1-yl)-10*H*-phenothiazine as a white powder (0.60 g, yield 73%).

10-(6-Hydroxyhexa-2,4-diyn-1-yl)-10*H*-phenothiazine 5-oxide (Gilman & Ranck, 1958)

To a solution of 10-(6-hydroxyhexa-2,4-diyn-1-yl)-10*H*-phenothiazine (0.04 g, 0.14 mmol) in ethanol (30 ml), hydrogen peroxide (0.2 ml, 3.9 mmol × 2) was added successively. Then, the solution was refluxed for 5 h. After the solvent was evaporated, the residue was extracted with dichloromethane (20 ml × 3) and washed with water (20 ml). After the organic layer was concentrated, the residue was purified by a column chromatography with dichloromethane/ethanol (50:1 *v*/*v*) as an eluent to give 10-(6-hydroxyhexa-2,4-diyn-1-yl)-10*H*-phenothiazine 5-oxide (0.03 g, yield 71%). The single crystals with sufficient quality for X-ray analysis were obtained by concentration of a chloroform solution.

### Refinement

The C-bound H atoms were placed at ideal positions and were treated as riding on their parent C atoms. The *U*_iso_(H) values of the H atoms were set at 1.2*U*_eq_(parent C atom). The O-bound H atoms were obtained from a difference Fourier map. The H13 atom was refined isotropically without any restrictions. The position of the H26 atom was refined with the restraint of O—H range between 0.82 Å and 0.86 Å. The *U*_iso_(H26) value was fixed at 1.5*U*_eq_ of O4.

### Crystal data

**Table:**

C_18_H_13_NO_2_S	*F*(000) = 1280.00
*M**_r_* = 307.37	*D*_x_ = 1.355 Mg m^−^^3^
Monoclinic, *P*2_1_/*c*	Mo *K*α radiation, λ = 0.71075 Å
Hall symbol: -P 2ybc	Cell parameters from 9306 reflections
*a* = 16.797 (5) Å	θ = 2.3–31.2°
*b* = 10.197 (3) Å	µ = 0.22 mm^−^^1^
*c* = 17.664 (5) Å	*T* = 93 K
β = 94.934 (5)°	Prism, colourless
*V* = 3014.3 (15) Å^3^	0.15 × 0.15 × 0.05 mm
*Z* = 8	

### Data collection

**Table:**

Rigaku Saturn724+ diffractometer	5523 reflections with *F*^2^ > 2σ(*F*^2^)
Detector resolution: 7.111 pixels mm^-1^	*R*_int_ = 0.033
ω scans	θ_max_ = 27.5°
Absorption correction: numerical (*NUMABS*; Rigaku, 1999)	*h* = −21→21
*T*_min_ = 0.969, *T*_max_ = 0.989	*k* = −13→11
24445 measured reflections	*l* = −22→22
6932 independent reflections	

### Refinement

**Table:**

Refinement on *F*^2^	Secondary atom site location: difference Fourier map
*R*[*F*^2^ > 2σ(*F*^2^)] = 0.050	Hydrogen site location: inferred from neighbouring sites
*wR*(*F*^2^) = 0.135	H atoms treated by a mixture of independent and constrained refinement
*S* = 1.08	*w* = 1/[σ^2^(*F*_o_^2^) + (0.0692*P*)^2^ + 0.9115*P*] where *P* = (*F*_o_^2^ + 2*F*_c_^2^)/3
6931 reflections	(Δ/σ)_max_ = 0.001
404 parameters	Δρ_max_ = 0.88 e Å^−^^3^
1 restraint	Δρ_min_ = −0.47 e Å^−^^3^
Primary atom site location: structure-invariant direct methods	

### Special details

**Table:**

Refinement. Refinement was performed using all reflections except for 1 with very negative *F*^2^. The weighted *R*-factor (*wR*) and goodness of fit (*S*) are based on *F*^2^. *R*-factor (gt) are based on *F*. The threshold expression of *F*^2^ > 2.0 σ(*F*^2^) is used only for calculating *R*-factor (gt).

### Fractional atomic coordinates and isotropic or equivalent isotropic displacement parameters (Å^2^)

**Table:**

	*x*	*y*	*z*	*U*_iso_*/*U*_eq_	
S1	0.30626 (3)	0.37416 (5)	0.39495 (3)	0.02511 (13)	
S2	0.16339 (3)	0.80322 (5)	0.61113 (3)	0.02991 (14)	
O1	0.36361 (8)	0.25896 (13)	0.40014 (8)	0.0299 (3)	
O2	−0.10191 (8)	0.61158 (15)	0.80031 (8)	0.0280 (3)	
O3	0.09579 (9)	0.90034 (15)	0.61077 (10)	0.0420 (4)	
O4	0.61087 (8)	0.67228 (15)	0.23764 (8)	0.0326 (4)	
N1	0.25753 (9)	0.34698 (15)	0.55932 (8)	0.0206 (4)	
N2	0.23463 (9)	0.90242 (15)	0.46339 (9)	0.0249 (4)	
C1	0.20387 (11)	0.29706 (17)	0.50186 (10)	0.0212 (4)	
C2	0.13522 (11)	0.22802 (18)	0.51913 (11)	0.0255 (4)	
C3	0.08004 (12)	0.18508 (19)	0.46171 (11)	0.0292 (5)	
C4	0.09045 (12)	0.20915 (19)	0.38558 (11)	0.0298 (5)	
C5	0.15870 (12)	0.27348 (18)	0.36725 (11)	0.0278 (5)	
C6	0.21564 (11)	0.31566 (17)	0.42466 (10)	0.0233 (4)	
C7	0.33286 (12)	0.47585 (18)	0.47324 (11)	0.0251 (4)	
C8	0.38433 (13)	0.5795 (2)	0.45994 (12)	0.0341 (5)	
C9	0.41471 (14)	0.6578 (3)	0.51915 (13)	0.0388 (6)	
C10	0.39262 (13)	0.6322 (2)	0.59173 (12)	0.0346 (5)	
C11	0.34146 (12)	0.53097 (19)	0.60589 (11)	0.0274 (4)	
C12	0.31011 (11)	0.44917 (17)	0.54639 (10)	0.0221 (4)	
C13	0.24625 (11)	0.31145 (18)	0.63824 (10)	0.0224 (4)	
C14	0.18691 (11)	0.39347 (18)	0.67257 (10)	0.0234 (4)	
C15	0.13874 (11)	0.46074 (18)	0.70120 (10)	0.0234 (4)	
C16	0.08236 (11)	0.53360 (18)	0.73504 (10)	0.0246 (4)	
C17	0.03417 (11)	0.59378 (18)	0.76754 (11)	0.0251 (4)	
C18	−0.02351 (11)	0.6651 (2)	0.81011 (12)	0.0286 (5)	
C19	0.18737 (11)	0.78941 (18)	0.45680 (11)	0.0244 (4)	
C20	0.16976 (12)	0.72862 (18)	0.38549 (11)	0.0273 (5)	
C21	0.12277 (12)	0.61690 (19)	0.37921 (12)	0.0286 (5)	
C22	0.09196 (12)	0.56089 (19)	0.44271 (12)	0.0294 (5)	
C23	0.10966 (12)	0.61816 (19)	0.51256 (12)	0.0286 (5)	
C24	0.15653 (11)	0.73151 (18)	0.52011 (11)	0.0255 (4)	
C25	0.25119 (11)	0.89494 (19)	0.60253 (11)	0.0266 (4)	
C26	0.29188 (12)	0.9335 (2)	0.67147 (12)	0.0298 (5)	
C27	0.35514 (12)	1.0200 (2)	0.67242 (12)	0.0328 (5)	
C28	0.37838 (12)	1.0661 (2)	0.60378 (13)	0.0327 (5)	
C29	0.34015 (12)	1.02790 (19)	0.53504 (12)	0.0290 (5)	
C30	0.27439 (11)	0.94121 (18)	0.53302 (11)	0.0248 (4)	
C31	0.25409 (12)	0.96831 (19)	0.39337 (11)	0.0278 (4)	
C32	0.31969 (12)	0.90391 (19)	0.35750 (11)	0.0282 (5)	
C33	0.37120 (12)	0.8436 (2)	0.32972 (11)	0.0280 (5)	
C34	0.42881 (12)	0.77033 (19)	0.29766 (11)	0.0277 (5)	
C35	0.47657 (12)	0.70260 (19)	0.26922 (11)	0.0272 (4)	
C36	0.53335 (11)	0.6173 (2)	0.23356 (12)	0.0287 (5)	
H1	0.1266	0.2107	0.5706	0.0306*	
H2	0.0342	0.1383	0.4745	0.0350*	
H3	0.0513	0.1818	0.3468	0.0358*	
H4	0.1670	0.2891	0.3155	0.0334*	
H5	0.3984	0.5960	0.4099	0.0410*	
H6	0.4501	0.7279	0.5105	0.0465*	
H7	0.4133	0.6860	0.6327	0.0415*	
H8	0.3273	0.5163	0.6561	0.0328*	
H9	0.2981	0.3191	0.6691	0.0269*	
H10	0.2291	0.2187	0.6398	0.0269*	
H11	−0.0049	0.6636	0.8648	0.0343*	
H12	−0.0254	0.7578	0.7934	0.0343*	
H13	−0.1010 (16)	0.542 (3)	0.8238 (16)	0.050 (8)*	
H14	0.1903	0.7646	0.3415	0.0328*	
H15	0.1113	0.5776	0.3308	0.0344*	
H16	0.0594	0.4847	0.4377	0.0353*	
H17	0.0898	0.5802	0.5563	0.0343*	
H18	0.2758	0.8998	0.7179	0.0357*	
H19	0.3822	1.0475	0.7191	0.0394*	
H20	0.4219	1.1257	0.6041	0.0393*	
H21	0.3581	1.0600	0.4890	0.0348*	
H22	0.2692	1.0603	0.4053	0.0333*	
H23	0.2059	0.9697	0.3569	0.0333*	
H24	0.5357	0.5310	0.2593	0.0344*	
H25	0.5142	0.6029	0.1796	0.0344*	
H26	0.6186 (16)	0.695 (3)	0.1926 (11)	0.0489*	

### Atomic displacement parameters (Å^2^)

**Table:**

	*U*^11^	*U*^22^	*U*^33^	*U*^12^	*U*^13^	*U*^23^
S1	0.0337 (3)	0.0229 (3)	0.0194 (3)	0.00180 (18)	0.00666 (19)	0.00054 (17)
S2	0.0313 (3)	0.0278 (3)	0.0311 (3)	−0.0024 (2)	0.0052 (2)	−0.0050 (2)
O1	0.0342 (8)	0.0273 (8)	0.0292 (7)	0.0066 (6)	0.0081 (6)	−0.0021 (6)
O2	0.0261 (7)	0.0278 (8)	0.0304 (8)	0.0005 (6)	0.0040 (6)	0.0057 (6)
O3	0.0294 (8)	0.0407 (9)	0.0559 (11)	−0.0002 (7)	0.0047 (7)	−0.0241 (8)
O4	0.0266 (8)	0.0436 (9)	0.0273 (8)	−0.0095 (7)	0.0008 (6)	0.0045 (7)
N1	0.0240 (8)	0.0213 (8)	0.0168 (7)	0.0002 (6)	0.0031 (6)	−0.0001 (6)
N2	0.0271 (8)	0.0201 (8)	0.0273 (8)	0.0003 (7)	0.0015 (7)	−0.0018 (7)
C1	0.0260 (9)	0.0176 (9)	0.0198 (9)	0.0040 (7)	0.0010 (7)	−0.0010 (7)
C2	0.0289 (10)	0.0254 (10)	0.0225 (9)	0.0017 (8)	0.0039 (8)	−0.0012 (8)
C3	0.0273 (10)	0.0264 (11)	0.0334 (11)	−0.0005 (8)	0.0002 (9)	−0.0041 (8)
C4	0.0326 (11)	0.0275 (11)	0.0280 (10)	0.0022 (9)	−0.0059 (9)	−0.0067 (8)
C5	0.0382 (11)	0.0230 (10)	0.0216 (9)	0.0084 (8)	−0.0010 (8)	−0.0037 (8)
C6	0.0307 (10)	0.0176 (9)	0.0216 (9)	0.0062 (8)	0.0029 (8)	−0.0004 (7)
C7	0.0315 (10)	0.0196 (10)	0.0246 (9)	0.0011 (8)	0.0052 (8)	0.0013 (8)
C8	0.0474 (13)	0.0270 (11)	0.0294 (11)	−0.0044 (9)	0.0110 (10)	0.0041 (9)
C9	0.0478 (14)	0.0278 (12)	0.0414 (13)	−0.0128 (10)	0.0075 (11)	−0.0005 (10)
C10	0.0398 (12)	0.0298 (12)	0.0336 (11)	−0.0058 (9)	0.0003 (10)	−0.0047 (9)
C11	0.0304 (10)	0.0276 (11)	0.0239 (10)	−0.0011 (8)	0.0018 (8)	−0.0020 (8)
C12	0.0234 (9)	0.0190 (9)	0.0242 (9)	0.0022 (7)	0.0031 (8)	0.0000 (7)
C13	0.0242 (9)	0.0266 (10)	0.0166 (8)	0.0022 (8)	0.0021 (7)	0.0020 (7)
C14	0.0256 (9)	0.0262 (10)	0.0182 (9)	−0.0027 (8)	0.0009 (8)	0.0013 (7)
C15	0.0286 (10)	0.0233 (10)	0.0184 (9)	−0.0027 (8)	0.0030 (8)	0.0003 (7)
C16	0.0277 (10)	0.0245 (10)	0.0217 (9)	−0.0012 (8)	0.0029 (8)	0.0015 (8)
C17	0.0286 (10)	0.0225 (10)	0.0244 (9)	−0.0015 (8)	0.0040 (8)	0.0021 (8)
C18	0.0275 (10)	0.0269 (11)	0.0324 (11)	0.0007 (8)	0.0089 (9)	−0.0027 (9)
C19	0.0223 (9)	0.0191 (9)	0.0313 (10)	0.0035 (7)	−0.0009 (8)	−0.0023 (8)
C20	0.0300 (10)	0.0230 (10)	0.0284 (10)	0.0052 (8)	−0.0010 (8)	−0.0006 (8)
C21	0.0312 (11)	0.0215 (10)	0.0324 (11)	0.0036 (8)	−0.0022 (9)	−0.0062 (8)
C22	0.0273 (10)	0.0185 (10)	0.0420 (12)	0.0001 (8)	0.0002 (9)	−0.0032 (9)
C23	0.0268 (10)	0.0238 (10)	0.0355 (11)	0.0002 (8)	0.0036 (9)	0.0013 (8)
C24	0.0242 (10)	0.0227 (10)	0.0296 (10)	0.0033 (8)	0.0013 (8)	−0.0037 (8)
C25	0.0259 (10)	0.0219 (10)	0.0317 (10)	0.0029 (8)	−0.0001 (8)	−0.0034 (8)
C26	0.0306 (11)	0.0282 (11)	0.0302 (10)	0.0071 (9)	0.0002 (9)	−0.0032 (9)
C27	0.0294 (11)	0.0300 (11)	0.0371 (12)	0.0065 (9)	−0.0087 (9)	−0.0051 (9)
C28	0.0268 (10)	0.0239 (11)	0.0461 (13)	−0.0006 (8)	−0.0048 (9)	−0.0044 (9)
C29	0.0263 (10)	0.0236 (10)	0.0364 (11)	0.0019 (8)	−0.0009 (9)	0.0002 (9)
C30	0.0252 (9)	0.0185 (9)	0.0298 (10)	0.0043 (8)	−0.0027 (8)	−0.0032 (8)
C31	0.0331 (11)	0.0193 (10)	0.0304 (10)	0.0027 (8)	−0.0000 (9)	0.0022 (8)
C32	0.0335 (11)	0.0228 (10)	0.0280 (10)	−0.0026 (8)	0.0012 (9)	0.0030 (8)
C33	0.0324 (11)	0.0256 (10)	0.0257 (10)	−0.0044 (9)	0.0016 (8)	0.0046 (8)
C34	0.0313 (11)	0.0273 (11)	0.0245 (10)	−0.0044 (8)	0.0022 (8)	0.0013 (8)
C35	0.0289 (10)	0.0270 (10)	0.0254 (10)	−0.0055 (8)	0.0009 (8)	0.0038 (8)
C36	0.0250 (10)	0.0285 (11)	0.0328 (11)	−0.0044 (8)	0.0040 (8)	−0.0010 (9)

### Geometric parameters (Å, º)

**Table:**

S1—O1	1.5169 (15)	C25—C26	1.400 (3)
S1—C6	1.757 (2)	C25—C30	1.402 (3)
S1—C7	1.755 (2)	C26—C27	1.380 (3)
S2—O3	1.5063 (17)	C27—C28	1.387 (4)
S2—C24	1.761 (2)	C28—C29	1.380 (3)
S2—C25	1.764 (2)	C29—C30	1.413 (3)
O2—C18	1.422 (3)	C31—C32	1.472 (3)
O4—C36	1.414 (3)	C32—C33	1.200 (3)
N1—C1	1.394 (3)	C33—C34	1.382 (3)
N1—C12	1.397 (3)	C34—C35	1.201 (3)
N1—C13	1.468 (3)	C35—C36	1.472 (3)
N2—C19	1.398 (3)	O2—H13	0.82 (3)
N2—C30	1.405 (3)	O4—H26	0.85 (2)
N2—C31	1.469 (3)	C2—H1	0.950
C1—C2	1.407 (3)	C3—H2	0.950
C1—C6	1.407 (3)	C4—H3	0.950
C2—C3	1.385 (3)	C5—H4	0.950
C3—C4	1.393 (3)	C8—H5	0.950
C4—C5	1.383 (3)	C9—H6	0.950
C5—C6	1.400 (3)	C10—H7	0.950
C7—C8	1.398 (3)	C11—H8	0.950
C7—C12	1.405 (3)	C13—H9	0.990
C8—C9	1.378 (3)	C13—H10	0.990
C9—C10	1.390 (4)	C18—H11	0.990
C10—C11	1.380 (3)	C18—H12	0.990
C11—C12	1.408 (3)	C20—H14	0.950
C13—C14	1.471 (3)	C21—H15	0.950
C14—C15	1.205 (3)	C22—H16	0.950
C15—C16	1.380 (3)	C23—H17	0.950
C16—C17	1.201 (3)	C26—H18	0.950
C17—C18	1.469 (3)	C27—H19	0.950
C19—C20	1.412 (3)	C28—H20	0.950
C19—C24	1.403 (3)	C29—H21	0.950
C20—C21	1.385 (3)	C31—H22	0.990
C21—C22	1.397 (3)	C31—H23	0.990
C22—C23	1.374 (3)	C36—H24	0.990
C23—C24	1.398 (3)	C36—H25	0.990
			
O1—S1—C6	106.32 (9)	C31—C32—C33	175.7 (2)
O1—S1—C7	107.01 (9)	C32—C33—C34	178.0 (3)
C6—S1—C7	97.68 (10)	C33—C34—C35	177.3 (3)
O3—S2—C24	106.20 (10)	C34—C35—C36	178.5 (2)
O3—S2—C25	106.65 (10)	O4—C36—C35	111.80 (17)
C24—S2—C25	97.53 (10)	C18—O2—H13	107.0 (18)
C1—N1—C12	122.09 (15)	C36—O4—H26	106.0 (17)
C1—N1—C13	118.22 (15)	C1—C2—H1	119.704
C12—N1—C13	118.30 (14)	C3—C2—H1	119.707
C19—N2—C30	121.91 (16)	C2—C3—H2	119.339
C19—N2—C31	118.21 (16)	C4—C3—H2	119.344
C30—N2—C31	119.01 (16)	C3—C4—H3	120.499
N1—C1—C2	121.03 (17)	C5—C4—H3	120.487
N1—C1—C6	121.34 (17)	C4—C5—H4	119.860
C2—C1—C6	117.62 (16)	C6—C5—H4	119.855
C1—C2—C3	120.59 (18)	C7—C8—H5	119.862
C2—C3—C4	121.32 (19)	C9—C8—H5	119.853
C3—C4—C5	119.01 (18)	C8—C9—H6	120.631
C4—C5—C6	120.28 (18)	C10—C9—H6	120.638
S1—C6—C1	122.19 (14)	C9—C10—H7	119.087
S1—C6—C5	116.32 (15)	C11—C10—H7	119.090
C1—C6—C5	121.07 (18)	C10—C11—H8	119.778
S1—C7—C8	115.68 (16)	C12—C11—H8	119.773
S1—C7—C12	122.63 (15)	N1—C13—H9	108.873
C8—C7—C12	121.46 (18)	N1—C13—H10	108.869
C7—C8—C9	120.3 (2)	C14—C13—H9	108.874
C8—C9—C10	118.7 (2)	C14—C13—H10	108.874
C9—C10—C11	121.8 (2)	H9—C13—H10	107.721
C10—C11—C12	120.45 (19)	O2—C18—H11	108.960
N1—C12—C7	121.36 (16)	O2—C18—H12	108.959
N1—C12—C11	121.39 (17)	C17—C18—H11	108.953
C7—C12—C11	117.25 (17)	C17—C18—H12	108.954
N1—C13—C14	113.48 (15)	H11—C18—H12	107.770
C13—C14—C15	179.47 (19)	C19—C20—H14	119.777
C14—C15—C16	177.9 (2)	C21—C20—H14	119.778
C15—C16—C17	177.0 (2)	C20—C21—H15	119.346
C16—C17—C18	177.8 (2)	C22—C21—H15	119.332
O2—C18—C17	113.11 (17)	C21—C22—H16	120.648
N2—C19—C20	120.65 (18)	C23—C22—H16	120.628
N2—C19—C24	121.76 (17)	C22—C23—H17	119.548
C20—C19—C24	117.58 (17)	C24—C23—H17	119.561
C19—C20—C21	120.45 (19)	C25—C26—H18	119.757
C20—C21—C22	121.32 (19)	C27—C26—H18	119.744
C21—C22—C23	118.72 (19)	C26—C27—H19	120.657
C22—C23—C24	120.9 (2)	C28—C27—H19	120.645
S2—C24—C19	123.61 (15)	C27—C28—H20	119.032
S2—C24—C23	114.99 (16)	C29—C28—H20	119.052
C19—C24—C23	121.02 (18)	C28—C29—H21	119.919
S2—C25—C26	115.02 (16)	C30—C29—H21	119.915
S2—C25—C30	123.47 (14)	N2—C31—H22	109.035
C26—C25—C30	121.04 (18)	N2—C31—H23	109.035
C25—C26—C27	120.5 (2)	C32—C31—H22	109.032
C26—C27—C28	118.70 (19)	C32—C31—H23	109.027
C27—C28—C29	121.92 (19)	H22—C31—H23	107.797
C28—C29—C30	120.2 (2)	O4—C36—H24	109.253
N2—C30—C25	121.68 (17)	O4—C36—H25	109.261
N2—C30—C29	120.66 (18)	C35—C36—H24	109.256
C25—C30—C29	117.66 (18)	C35—C36—H25	109.256
N2—C31—C32	112.79 (16)	H24—C36—H25	107.939
			
O1—S1—C6—C1	77.83 (14)	C2—C1—C6—C5	3.6 (3)
O1—S1—C6—C5	−94.80 (13)	C6—C1—C2—C3	−2.6 (3)
O1—S1—C7—C8	94.67 (14)	C1—C2—C3—C4	−0.3 (3)
O1—S1—C7—C12	−79.86 (16)	C2—C3—C4—C5	2.2 (3)
C6—S1—C7—C8	−155.59 (13)	C3—C4—C5—C6	−1.1 (3)
C6—S1—C7—C12	29.88 (16)	C4—C5—C6—S1	170.93 (15)
C7—S1—C6—C1	−32.48 (15)	C4—C5—C6—C1	−1.8 (3)
C7—S1—C6—C5	154.89 (12)	S1—C7—C8—C9	−174.02 (13)
O3—S2—C24—C19	82.48 (15)	S1—C7—C12—N1	−6.9 (3)
O3—S2—C24—C23	−90.47 (14)	S1—C7—C12—C11	174.06 (12)
O3—S2—C25—C26	90.62 (14)	C8—C7—C12—N1	178.84 (17)
O3—S2—C25—C30	−81.60 (16)	C8—C7—C12—C11	−0.2 (3)
C24—S2—C25—C26	−159.91 (13)	C12—C7—C8—C9	0.6 (3)
C24—S2—C25—C30	27.87 (16)	C7—C8—C9—C10	−0.5 (3)
C25—S2—C24—C19	−27.36 (16)	C8—C9—C10—C11	0.1 (4)
C25—S2—C24—C23	159.69 (12)	C9—C10—C11—C12	0.3 (3)
C1—N1—C12—C7	−23.1 (3)	C10—C11—C12—N1	−179.29 (17)
C1—N1—C12—C11	155.82 (15)	C10—C11—C12—C7	−0.3 (3)
C12—N1—C1—C2	−158.65 (15)	N2—C19—C20—C21	−179.84 (15)
C12—N1—C1—C6	20.3 (3)	N2—C19—C24—S2	7.7 (3)
C1—N1—C13—C14	−82.95 (19)	N2—C19—C24—C23	−179.71 (15)
C13—N1—C1—C2	7.7 (3)	C20—C19—C24—S2	−172.93 (15)
C13—N1—C1—C6	−173.35 (14)	C20—C19—C24—C23	−0.4 (3)
C12—N1—C13—C14	83.93 (19)	C24—C19—C20—C21	0.8 (3)
C13—N1—C12—C7	170.51 (14)	C19—C20—C21—C22	−0.4 (3)
C13—N1—C12—C11	−10.5 (3)	C20—C21—C22—C23	−0.6 (3)
C19—N2—C30—C25	−19.6 (3)	C21—C22—C23—C24	1.0 (3)
C19—N2—C30—C29	159.75 (15)	C22—C23—C24—S2	172.62 (16)
C30—N2—C19—C20	−159.11 (15)	C22—C23—C24—C19	−0.5 (3)
C30—N2—C19—C24	20.2 (3)	S2—C25—C26—C27	−171.30 (13)
C19—N2—C31—C32	−79.65 (19)	S2—C25—C30—N2	−8.9 (3)
C31—N2—C19—C20	10.0 (3)	S2—C25—C30—C29	171.77 (12)
C31—N2—C19—C24	−170.65 (15)	C26—C25—C30—N2	179.37 (16)
C30—N2—C31—C32	89.82 (19)	C26—C25—C30—C29	−0.0 (3)
C31—N2—C30—C25	171.34 (15)	C30—C25—C26—C27	1.1 (3)
C31—N2—C30—C29	−9.3 (3)	C25—C26—C27—C28	−1.1 (3)
N1—C1—C2—C3	176.43 (15)	C26—C27—C28—C29	−0.0 (3)
N1—C1—C6—S1	12.3 (3)	C27—C28—C29—C30	1.1 (3)
N1—C1—C6—C5	−175.40 (14)	C28—C29—C30—N2	179.52 (17)
C2—C1—C6—S1	−168.70 (14)	C28—C29—C30—C25	−1.1 (3)

### Hydrogen-bond geometry (Å, º)

**Table:**

*D*—H···*A*	*D*—H	H···*A*	*D*···*A*	*D*—H···*A*
O2—H13···O3^i^	0.82 (3)	1.85 (3)	2.663 (3)	172 (3)
O4—H26···O1^ii^	0.85 (2)	1.81 (2)	2.659 (3)	175 (3)

Symmetry codes: (i) −*x*, *y*−1/2, −*z*+3/2; (ii) −*x*+1, *y*+1/2, −*z*+1/2.
